# Supplementary figures and images for: The Axin scaffold protects the kinase GSK3β from cross-pathway inhibition
Source: eLife. 2023 Aug 7;12:e85444. doi: 10.7554/eLife.85444 (PMC10442075; doi:10.7554/eLife.85444)

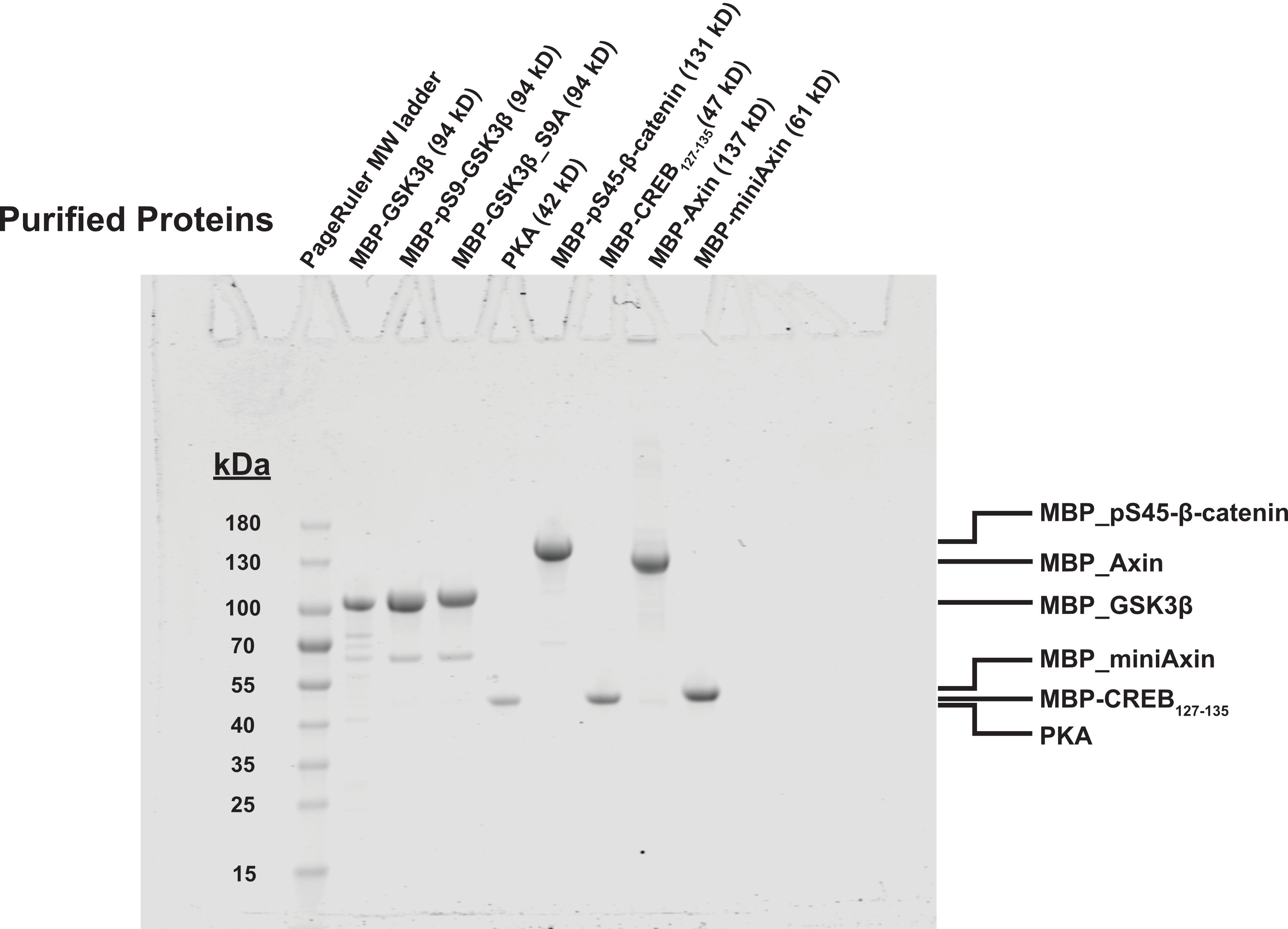

Supplement: Figure 2—figure supplement 1—source data 1. — Coomassie-stained SDS-PAGE of purified proteins used in this work. All proteins except PKA were purified as MBP fusion proteins (see Materials and methods). Unphosphorylated GSK3β was purified after coexpression with lambda phosphatase (see Materials and methods). pS45-β-catenin was purified after coexpression with CK1α as described previously (Gavagan et al., 2020). Phosphorylated GSK3β and GSK3β_S9A were purified after in vitro phosphorylation with PKA (see Materials and methods). Each lane was loaded with 10 μL of 4 μM protein. [file elife-85444-fig2-figsupp1-data1.zip › Figure 2-figure supplement 1 - source data 1/Figure 2-figure supplement 1 - source data 1 labeled.png]

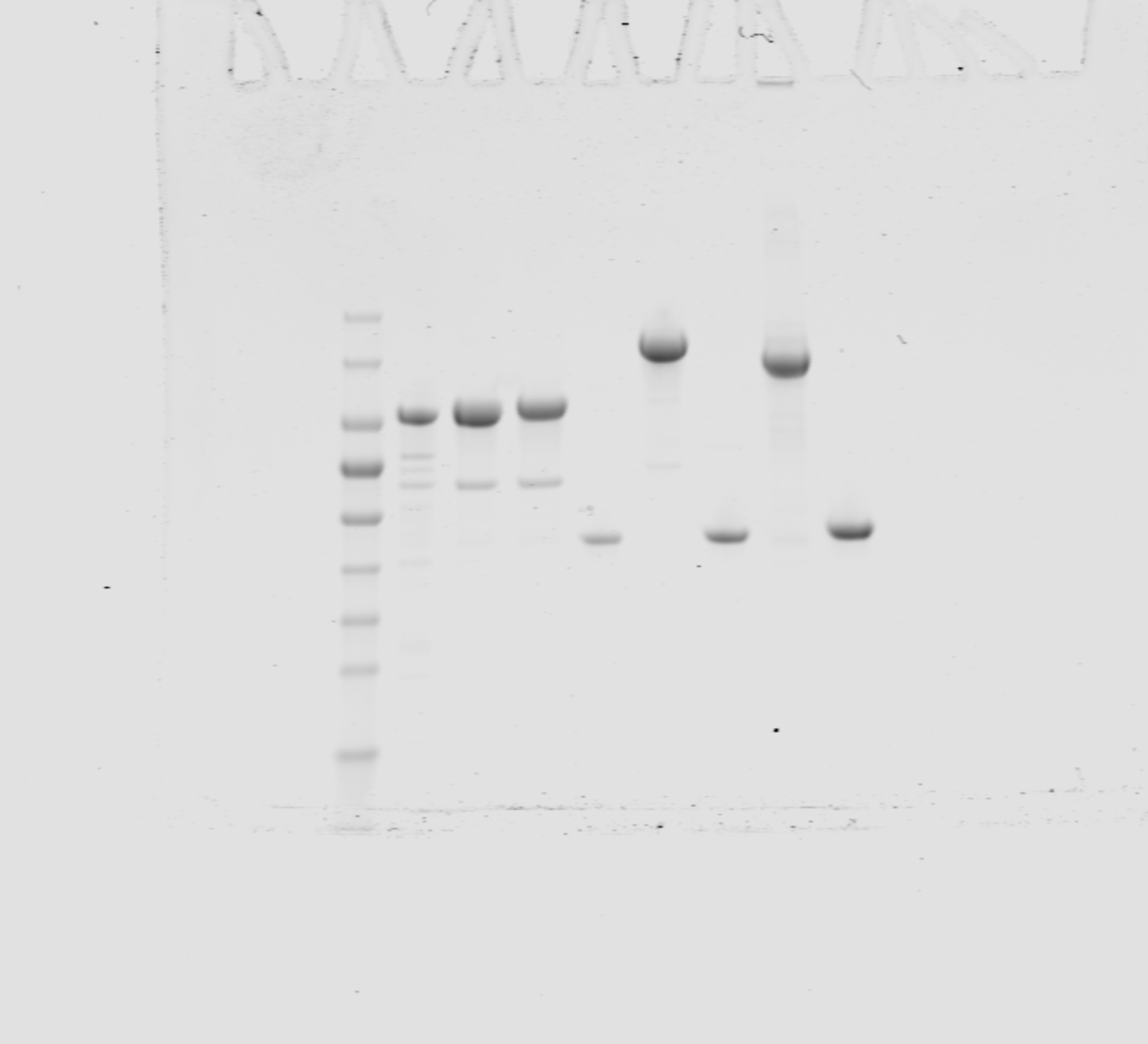

Supplement: Figure 2—figure supplement 1—source data 1. — Coomassie-stained SDS-PAGE of purified proteins used in this work. All proteins except PKA were purified as MBP fusion proteins (see Materials and methods). Unphosphorylated GSK3β was purified after coexpression with lambda phosphatase (see Materials and methods). pS45-β-catenin was purified after coexpression with CK1α as described previously (Gavagan et al., 2020). Phosphorylated GSK3β and GSK3β_S9A were purified after in vitro phosphorylation with PKA (see Materials and methods). Each lane was loaded with 10 μL of 4 μM protein. [file elife-85444-fig2-figsupp1-data1.zip › Figure 2-figure supplement 1 - source data 1/Figure 2-figure supplement 1 - source data 1 raw.tif]

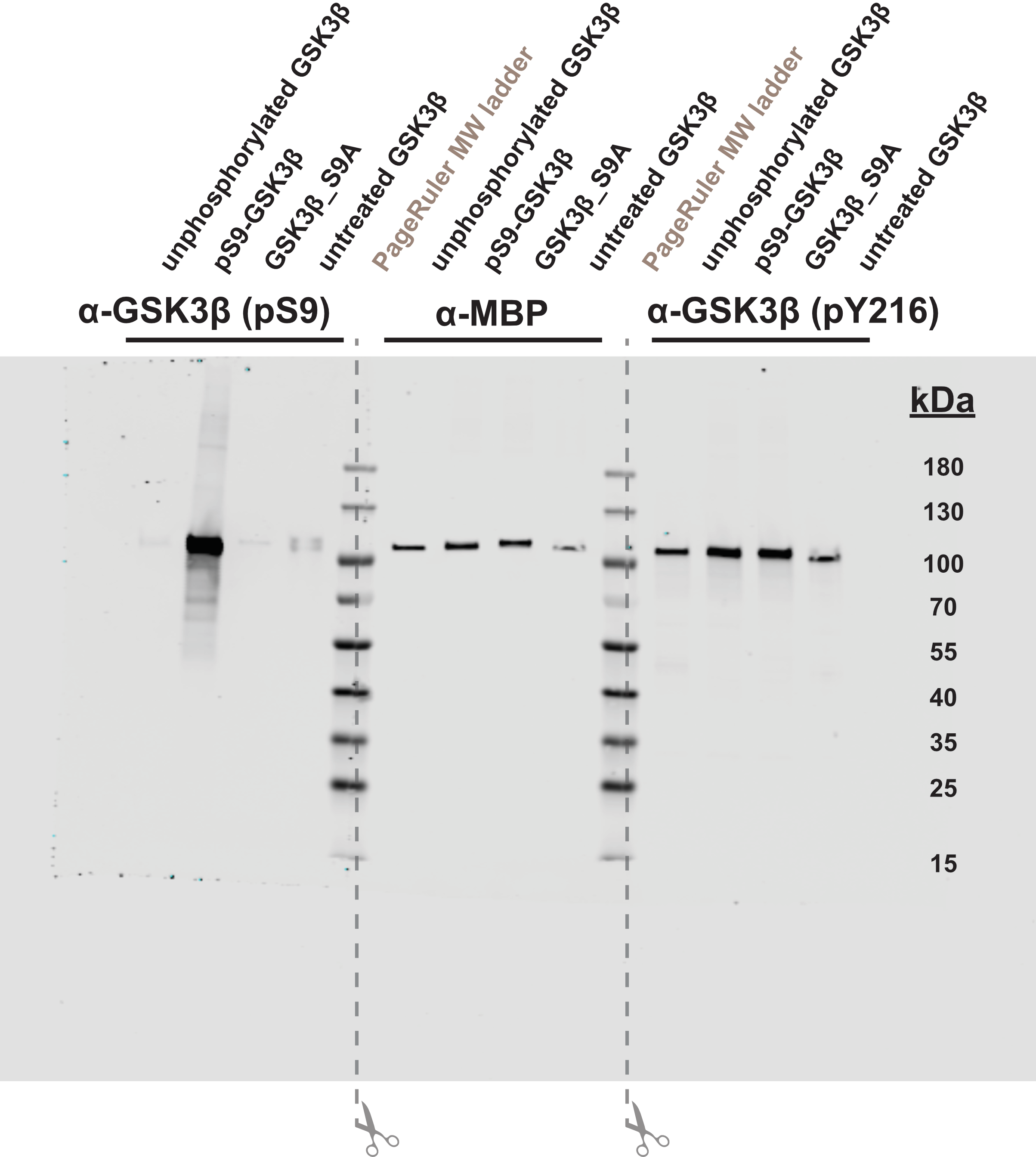

Supplement: Figure 2—figure supplement 1—source data 2. — Western blot for phosphorylation state of GSK3β at Ser9 and Tyr216. GSK3β samples are unphosphorylated GSK3β, pS9-GSK3β, GSK3β_S9A, and untreated GSK3β (unmodified recombinant protein, not coexpressed with lambda phosphatase or treated with PKA). After the western blot transfer, the membrane was cut down the center of the MW ladder lanes so each third of the membrane could be incubated with separate antibodies (α-pS9-GSK3β, α-MBP for total protein, and α-pY216-GSK3β). The membrane fragments were placed back together for imaging. [file elife-85444-fig2-figsupp1-data2.zip › Figure 2-figure supplement 1 - source data 2/Figure 2-figure supplement 1 - source data 2 labeled.png]

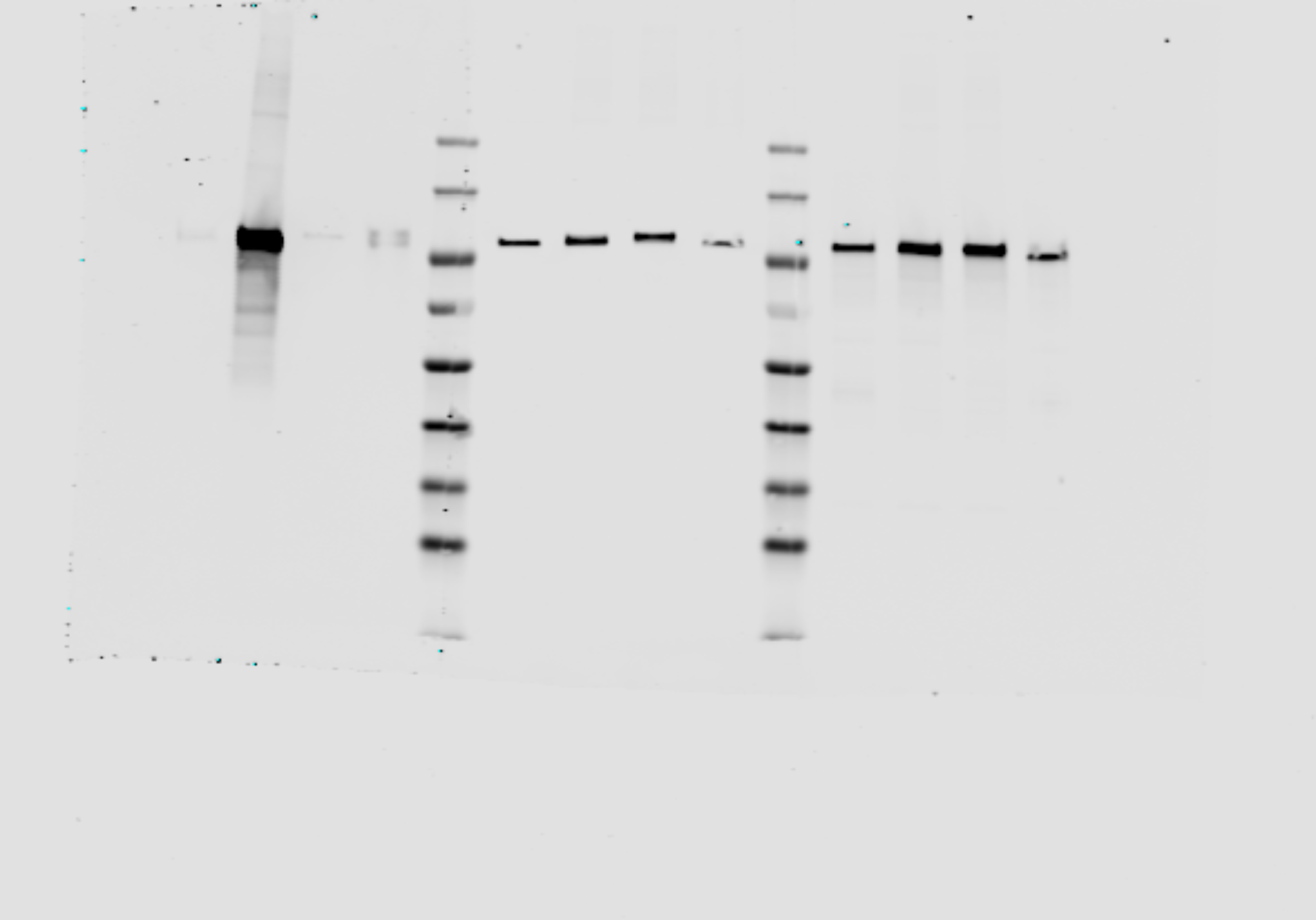

Supplement: Figure 2—figure supplement 1—source data 2. — Western blot for phosphorylation state of GSK3β at Ser9 and Tyr216. GSK3β samples are unphosphorylated GSK3β, pS9-GSK3β, GSK3β_S9A, and untreated GSK3β (unmodified recombinant protein, not coexpressed with lambda phosphatase or treated with PKA). After the western blot transfer, the membrane was cut down the center of the MW ladder lanes so each third of the membrane could be incubated with separate antibodies (α-pS9-GSK3β, α-MBP for total protein, and α-pY216-GSK3β). The membrane fragments were placed back together for imaging. [file elife-85444-fig2-figsupp1-data2.zip › Figure 2-figure supplement 1 - source data 2/Figure 2-figure supplement 1 - source data 2 raw.tif]

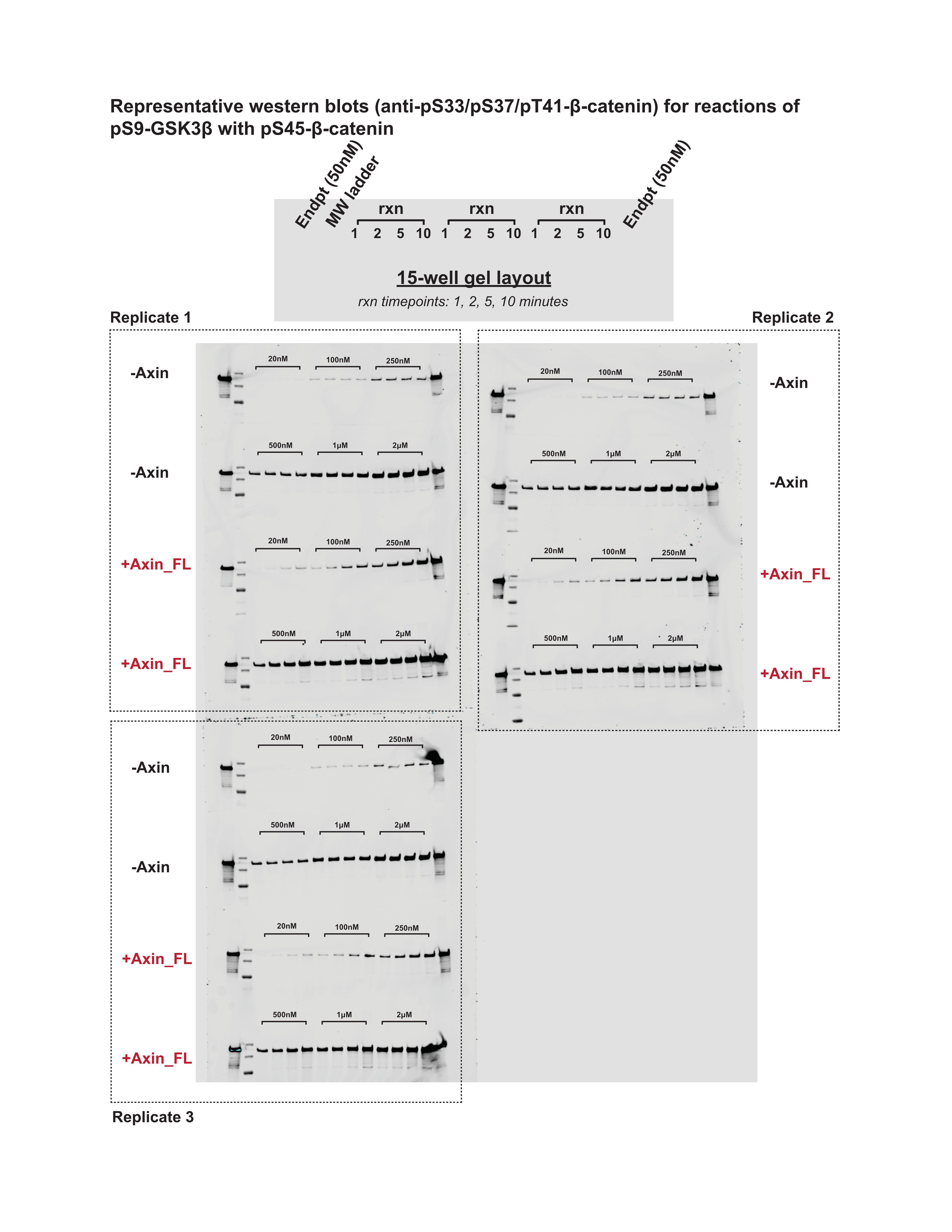

Supplement: Figure 2—figure supplement 2—source data 1. — Western blots for reactions of varying concentrations of pS45-β-catenin with 10 nM pS9-GSK3β in the presence and absence of 500 nM Axin. All gel samples were diluted 1:5 to prevent a gel smearing artifact (see Materials and methods). [file elife-85444-fig2-figsupp2-data1.zip › Figure 2-figure supplement 2 - source data 1/Figure 2-figure supplement 2 - source data 1 labeled.png]

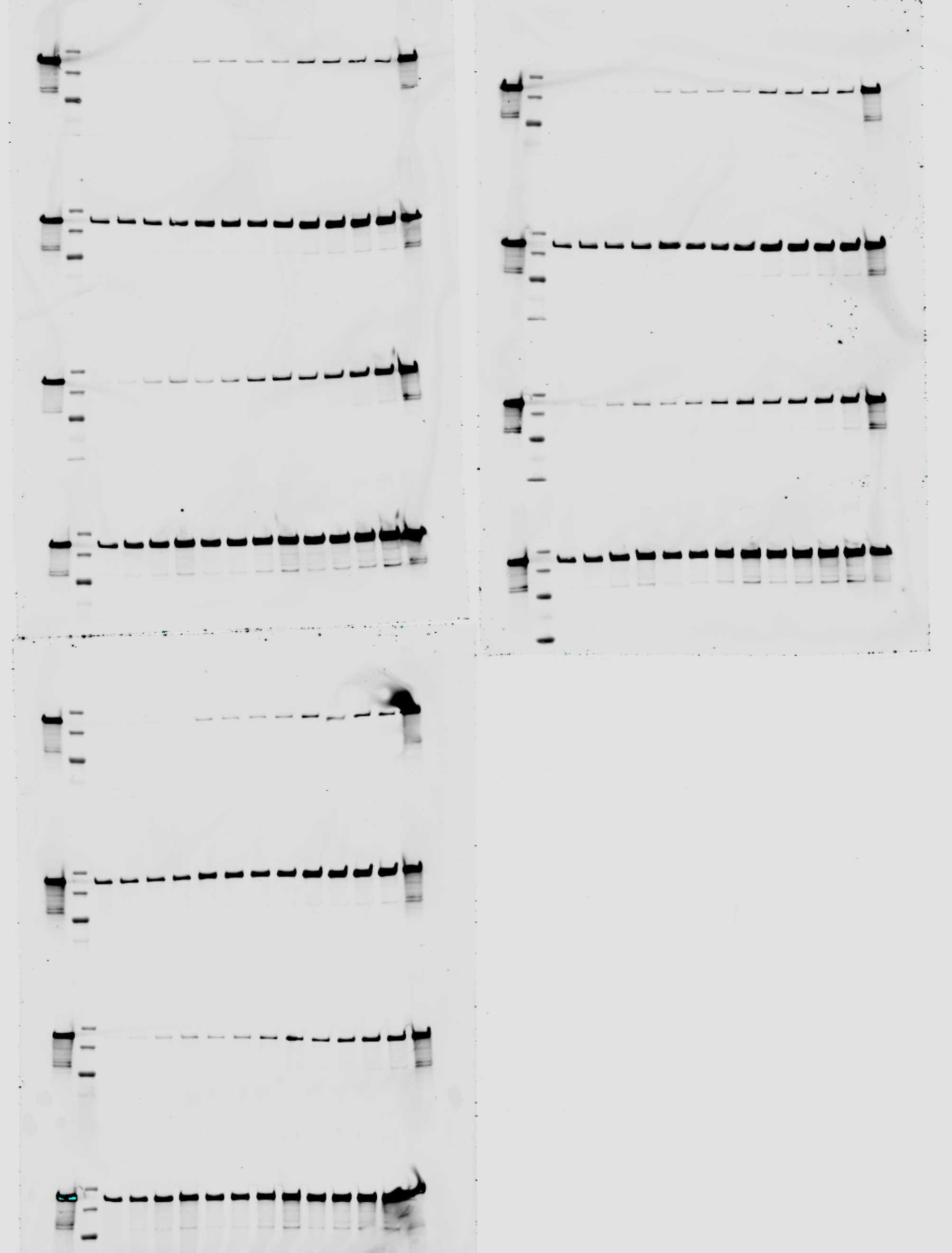

Supplement: Figure 2—figure supplement 2—source data 1. — Western blots for reactions of varying concentrations of pS45-β-catenin with 10 nM pS9-GSK3β in the presence and absence of 500 nM Axin. All gel samples were diluted 1:5 to prevent a gel smearing artifact (see Materials and methods). [file elife-85444-fig2-figsupp2-data1.zip › Figure 2-figure supplement 2 - source data 1/Figure 2-figure supplement 2 - source data 1 raw.tif]

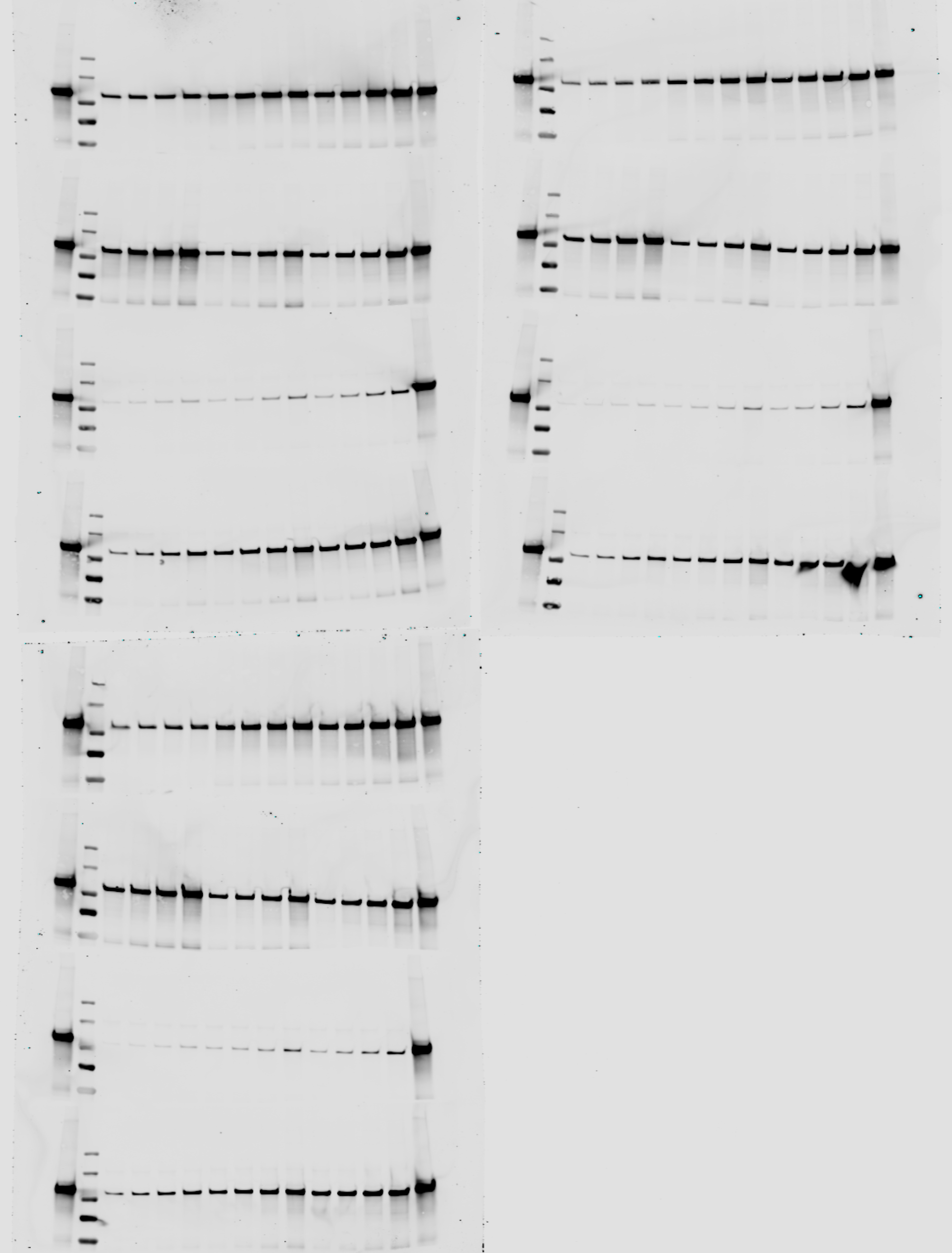

Supplement: Figure 2—figure supplement 2—source data 2. — Western blots for reactions of varying concentrations of GSK3β with 20 nM PKA +/-500 nM Axin. 500 nM GSK3β reaction gel samples without Axin were diluted 1:4 to prevent overloading the gel; all other reactions were diluted 1:2 (see Materials and methods). [file elife-85444-fig2-figsupp2-data2.zip › Figure 2-figure supplement 2 - source data 2/Figure 2-figure supplement 2 - source data 2 raw.tif]

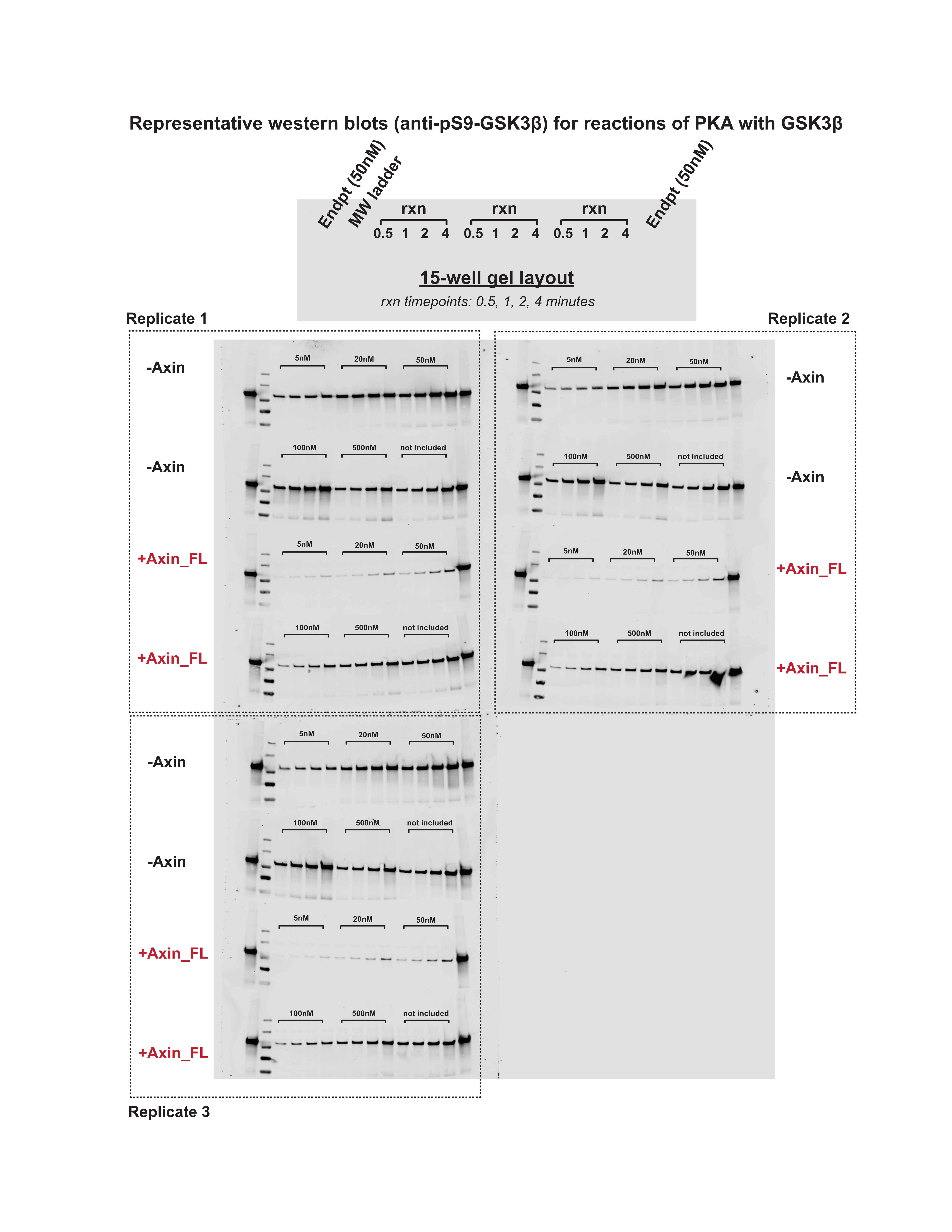

Supplement: Figure 2—figure supplement 2—source data 2. — Western blots for reactions of varying concentrations of GSK3β with 20 nM PKA +/-500 nM Axin. 500 nM GSK3β reaction gel samples without Axin were diluted 1:4 to prevent overloading the gel; all other reactions were diluted 1:2 (see Materials and methods). [file elife-85444-fig2-figsupp2-data2.zip › Figure 2-figure supplement 2 - source data 2/Figure 2-figure supplement 2 - source data 2 labeled.png]

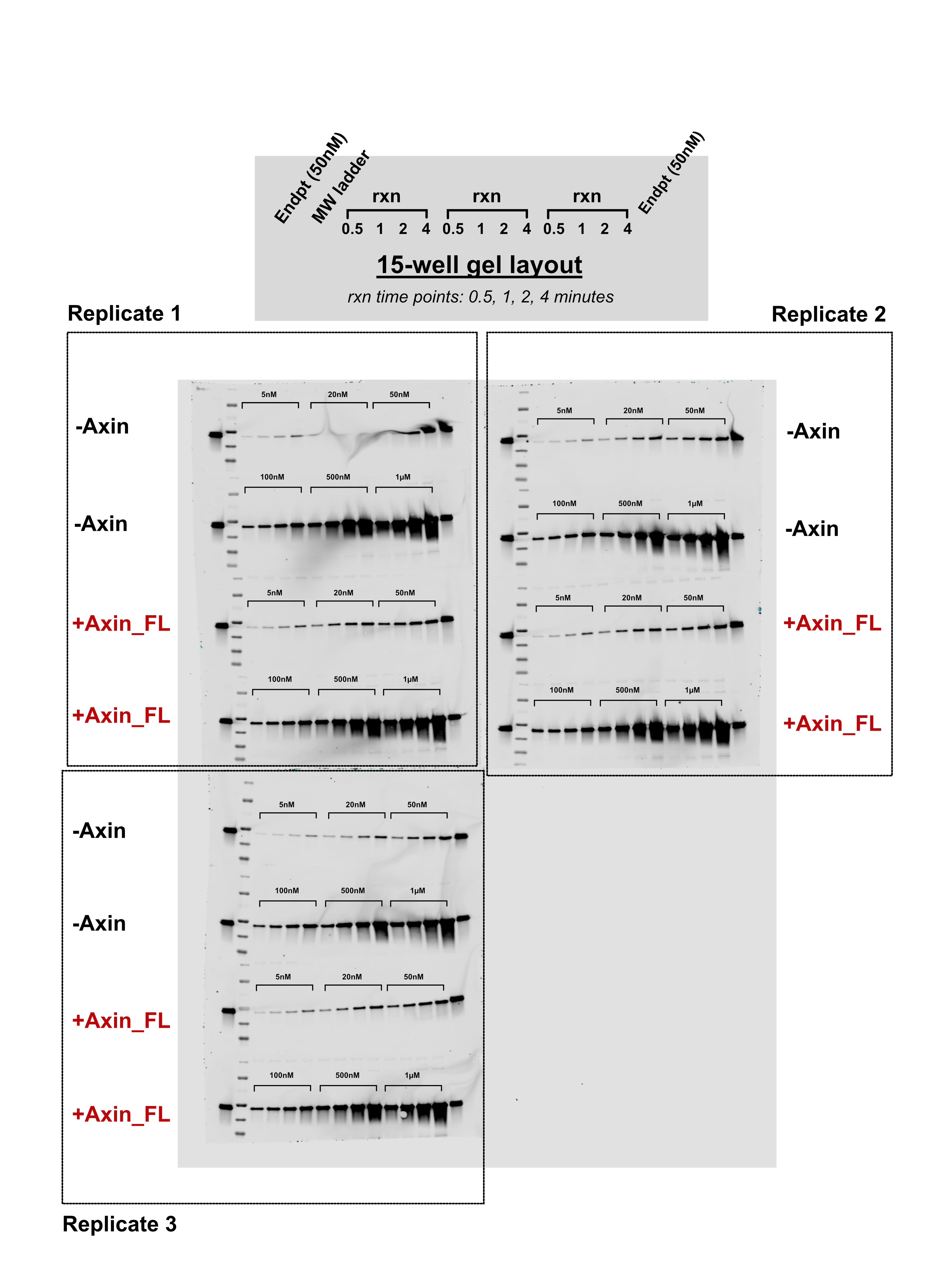

Supplement: Figure 2—figure supplement 2—source data 3. — Western blots for reactions of varying concentrations of CREB127-135 with 20 nM PKA in the presence and absence of 500 nM Axin. [file elife-85444-fig2-figsupp2-data3.zip › Figure 2-figure supplement 2 - source data 3/Figure 2-figure supplement 2 - source data 3 labeled.png]

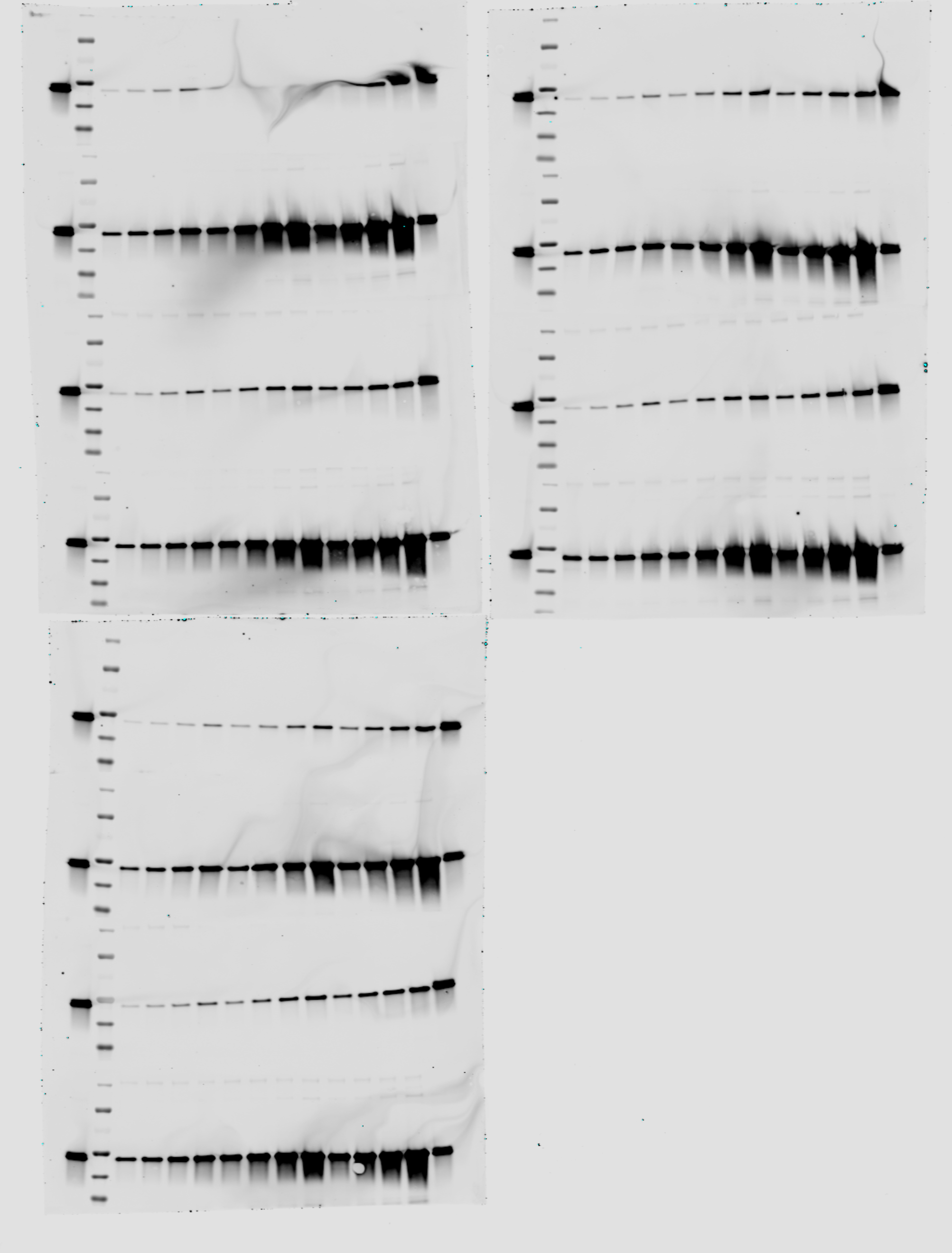

Supplement: Figure 2—figure supplement 2—source data 3. — Western blots for reactions of varying concentrations of CREB127-135 with 20 nM PKA in the presence and absence of 500 nM Axin. [file elife-85444-fig2-figsupp2-data3.zip › Figure 2-figure supplement 2 - source data 3/Figure 2-figure supplement 2 - source data 3 raw.tif]

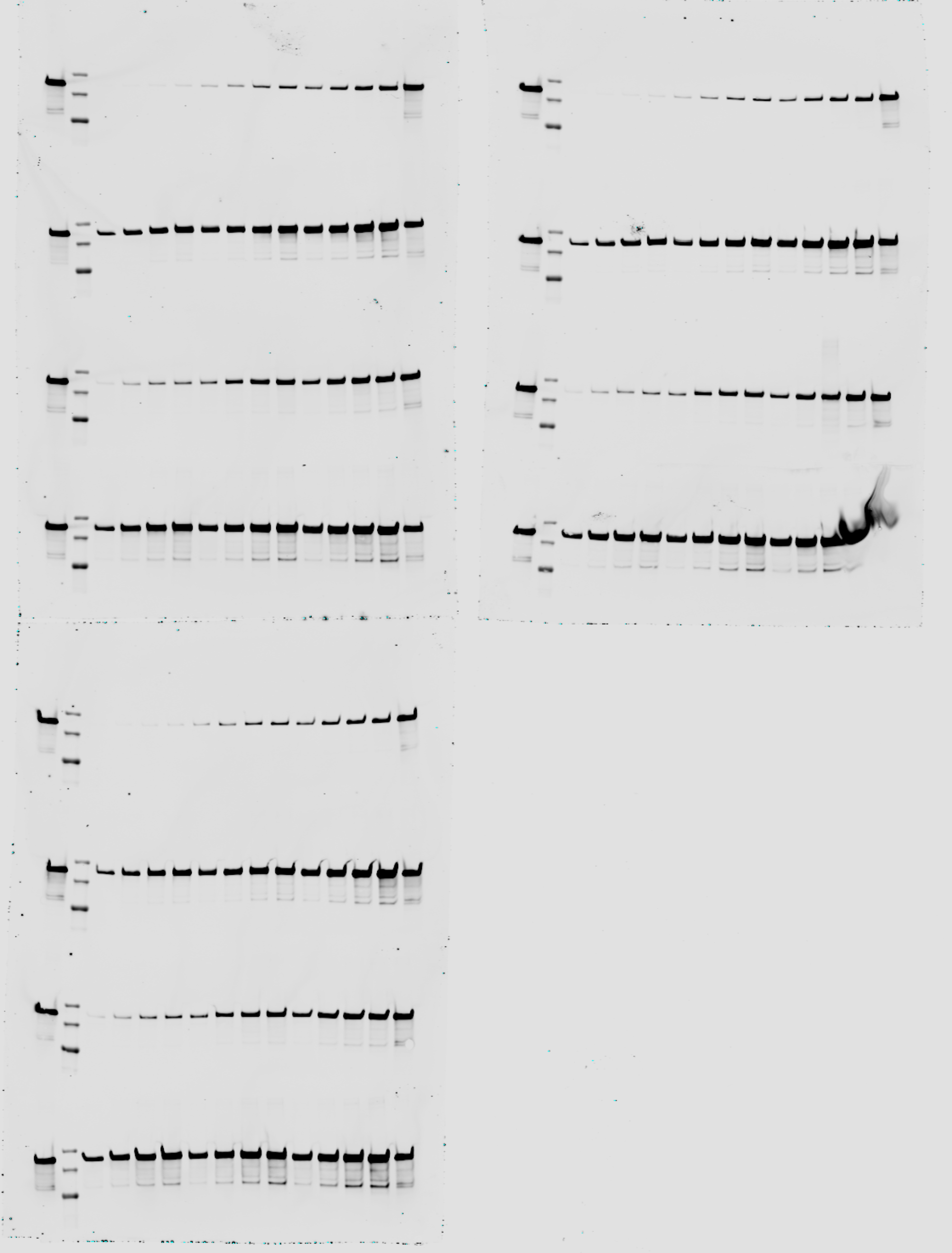

Supplement: Figure 2—figure supplement 4—source data 1. — Western blots for reactions of varying concentrations of pS45-β-catenin with 10 nM GSK3β in the presence and absence of 500 nM Axin. All gel samples were diluted 1:5 to prevent a gel smearing artifact (see Materials and methods). [file elife-85444-fig2-figsupp4-data1.zip › Figure 2-figure supplement 4 - source data/Figure 2-figure supplement 4 - source data raw.tif]

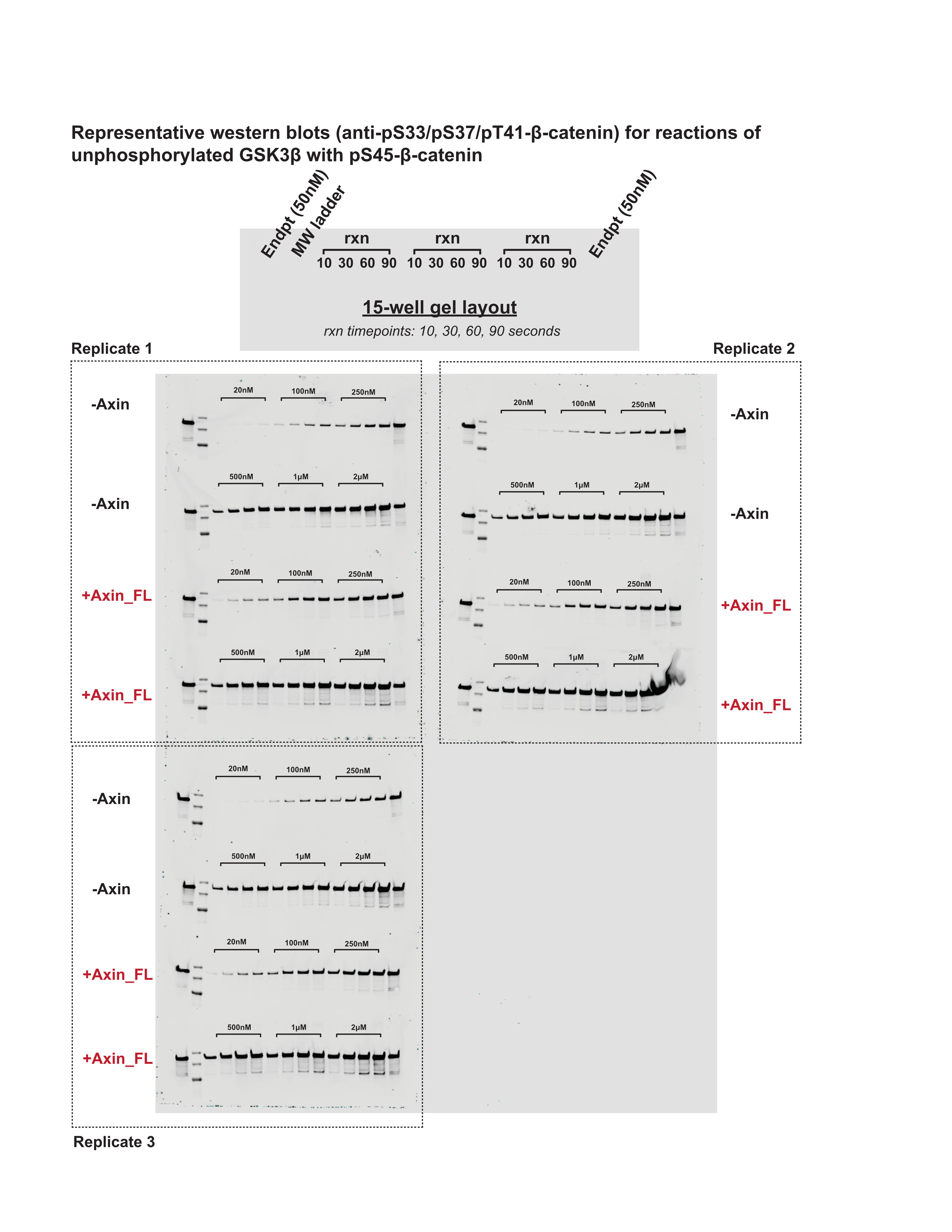

Supplement: Figure 2—figure supplement 4—source data 1. — Western blots for reactions of varying concentrations of pS45-β-catenin with 10 nM GSK3β in the presence and absence of 500 nM Axin. All gel samples were diluted 1:5 to prevent a gel smearing artifact (see Materials and methods). [file elife-85444-fig2-figsupp4-data1.zip › Figure 2-figure supplement 4 - source data/Figure 2-figure supplement 4 - source data labeled.png]

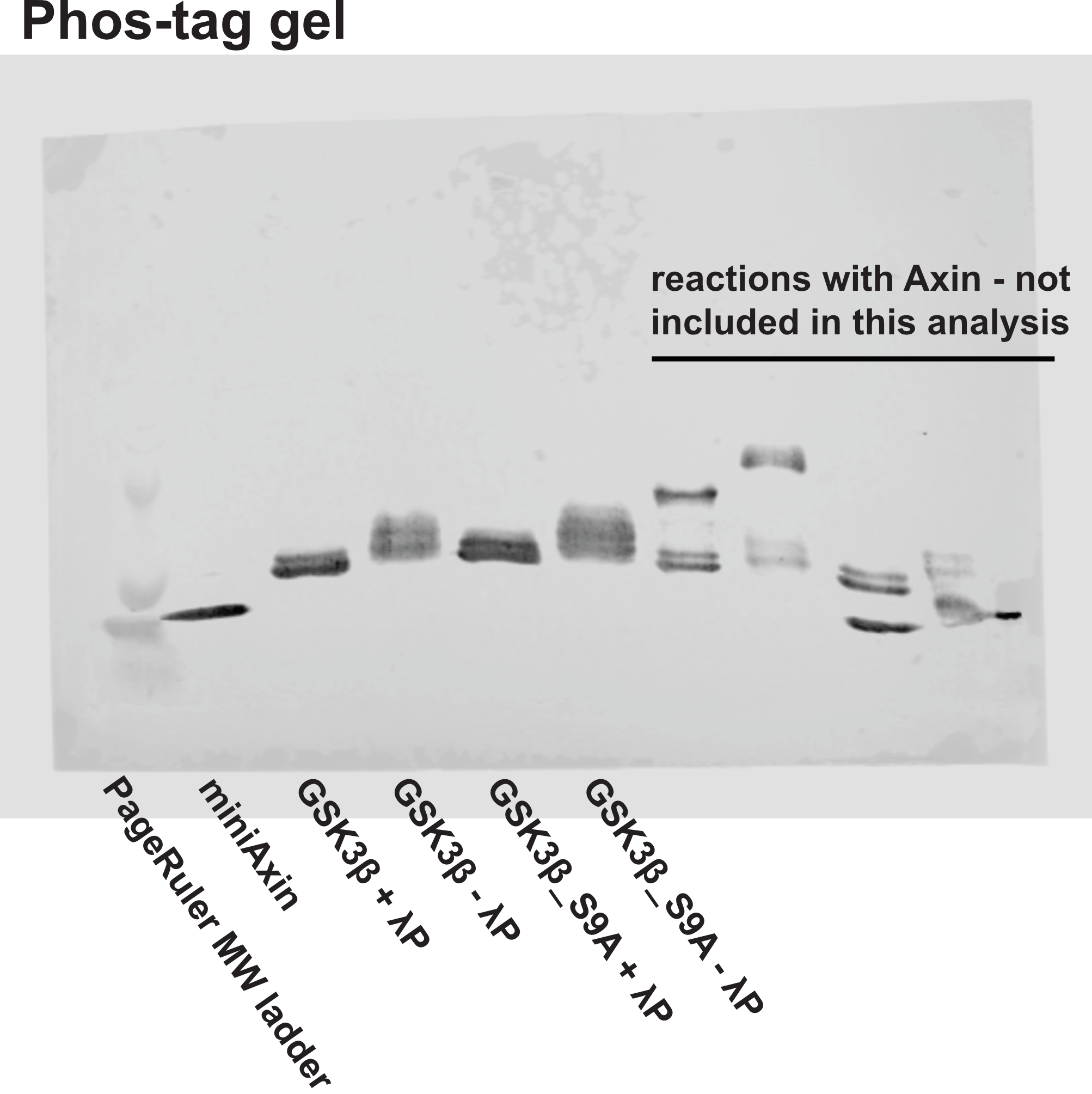

Supplement: Figure 2—figure supplement 9—source data 1. — Recombinant GSK3β is phosphorylated at phosphosites other than Ser9. Phos-tag gel of GSK3β and GSK3β_S9A with and without treatment with lambda phosphatase. Phos-tag gels were prepared and run as previously described (Gavagan et al., 2020; see Materials and methods). Samples with GSK3β or GSK3β_S9A were prepared in PMP buffer (NEB) with 1 mM MnCl2 and 400 nM GSK3β or GSK3β_S9A and incubated in the presence or absence of 30 μM lambda phosphatase for 30 min at 30 °C. The slower-migrating species are phosphorylated GSK3β or GSK3β_S9A. The presence of phosphorylated bands in GSK3β_S9A indicates that additional sites besides Ser9 are phosphorylated in recombinant GSK3β. [file elife-85444-fig2-figsupp9-data1.zip › Figure 2-figure supplement 9 - source data/Figure 2-figure supplement 9 - source data labeled.png]

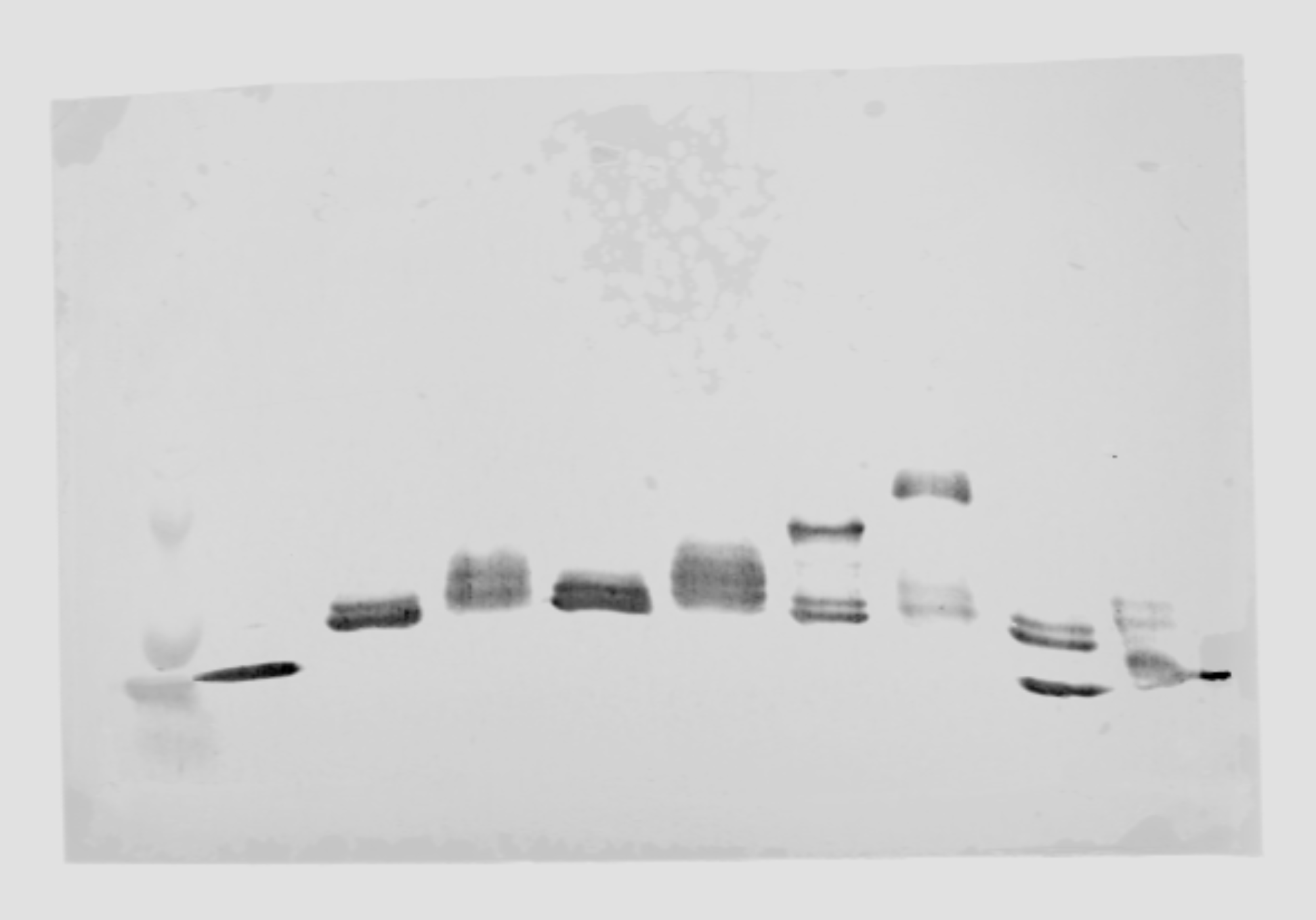

Supplement: Figure 2—figure supplement 9—source data 1. — Recombinant GSK3β is phosphorylated at phosphosites other than Ser9. Phos-tag gel of GSK3β and GSK3β_S9A with and without treatment with lambda phosphatase. Phos-tag gels were prepared and run as previously described (Gavagan et al., 2020; see Materials and methods). Samples with GSK3β or GSK3β_S9A were prepared in PMP buffer (NEB) with 1 mM MnCl2 and 400 nM GSK3β or GSK3β_S9A and incubated in the presence or absence of 30 μM lambda phosphatase for 30 min at 30 °C. The slower-migrating species are phosphorylated GSK3β or GSK3β_S9A. The presence of phosphorylated bands in GSK3β_S9A indicates that additional sites besides Ser9 are phosphorylated in recombinant GSK3β. [file elife-85444-fig2-figsupp9-data1.zip › Figure 2-figure supplement 9 - source data/Figure 2-figure supplement 9 - source data raw.tif]

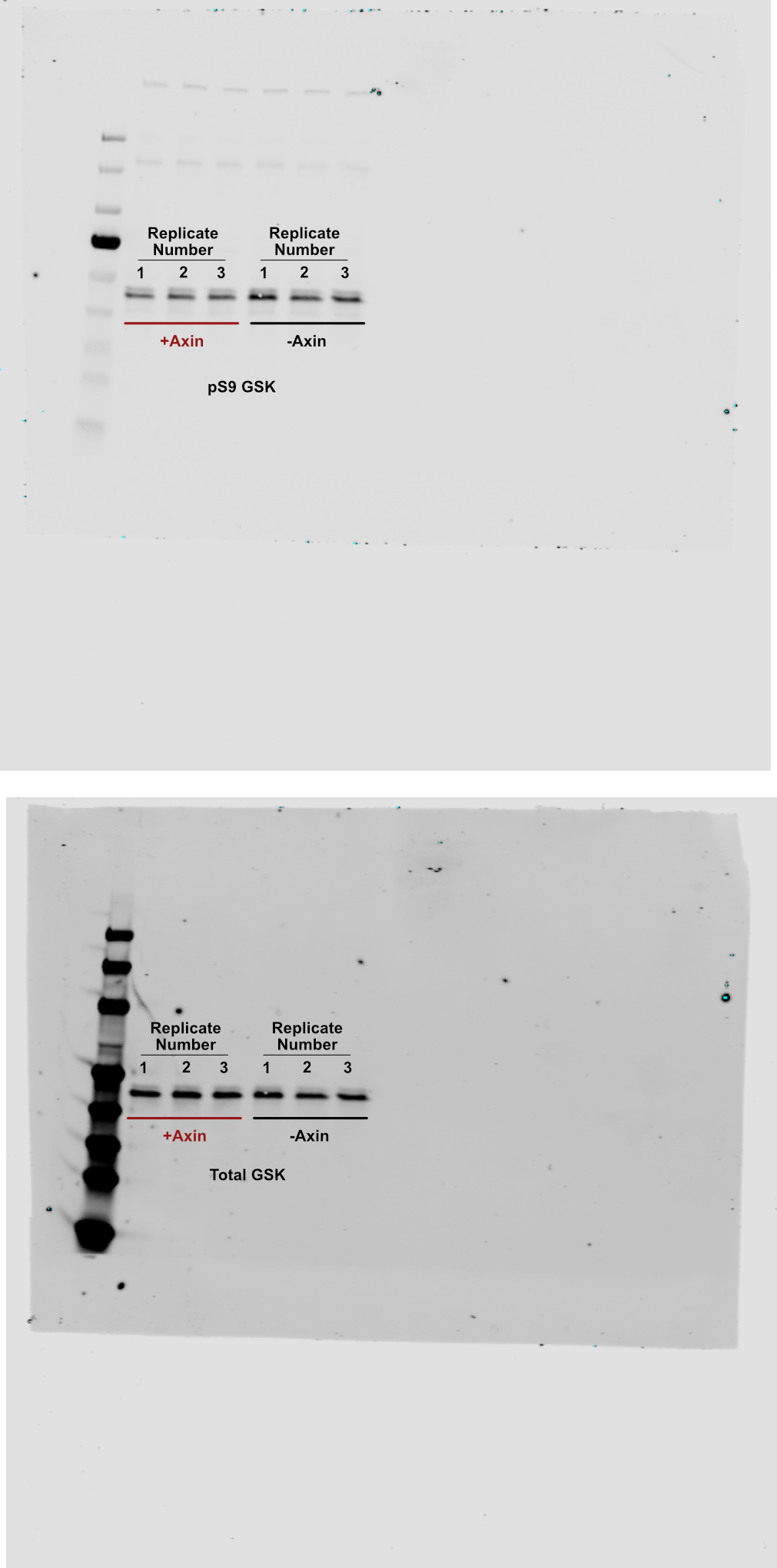

Supplement: Figure 4—source data 2. — Western blot images of pS9-GSK3β and total GSK3β in HEK293 cells transiently expressing Axin or a negative control, related to Figure 4C. [file elife-85444-fig4-data2.zip › Figure 4 - source data 2/Figure 4C - source data labeled.tiff]

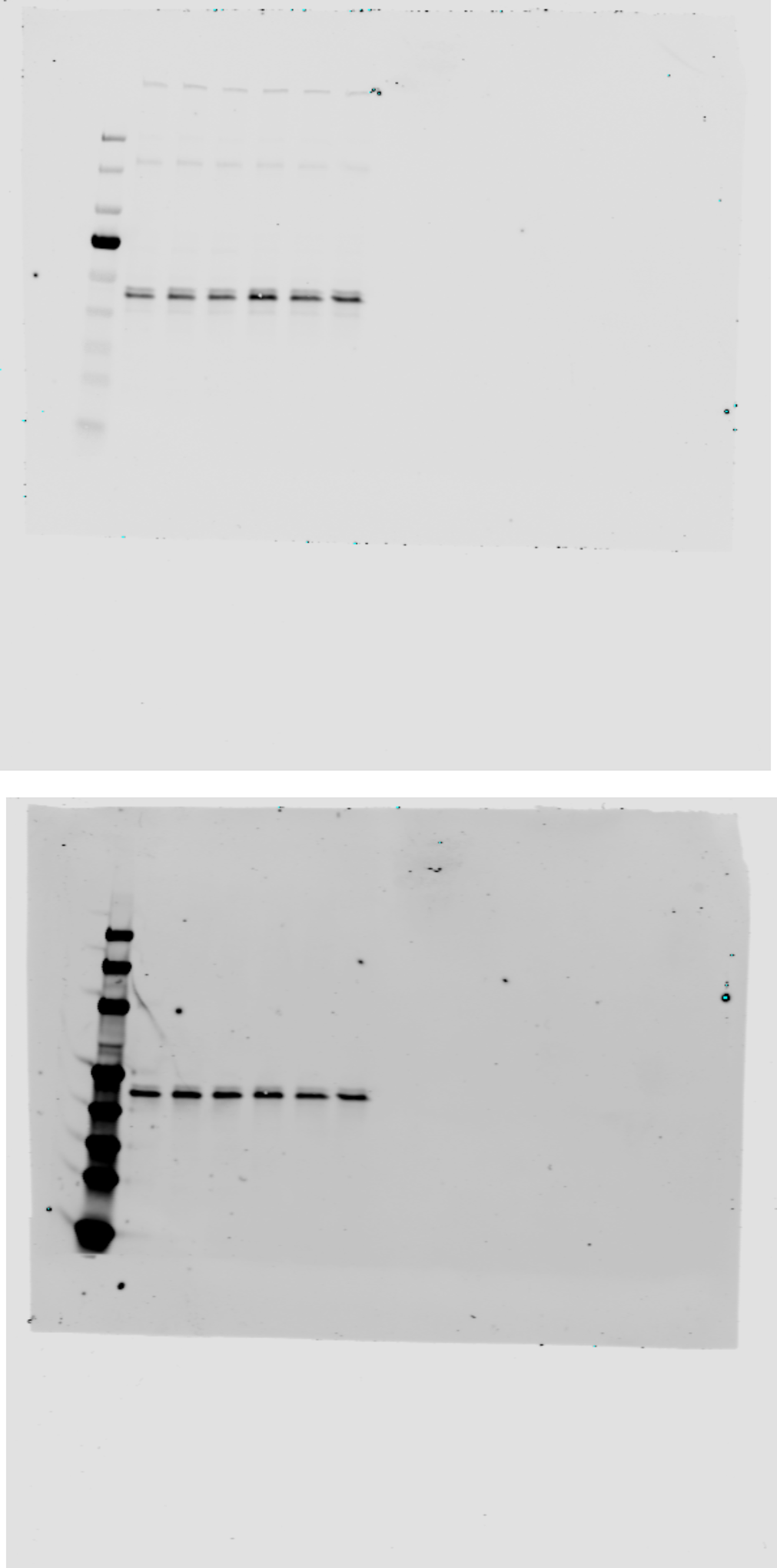

Supplement: Figure 4—source data 2. — Western blot images of pS9-GSK3β and total GSK3β in HEK293 cells transiently expressing Axin or a negative control, related to Figure 4C. [file elife-85444-fig4-data2.zip › Figure 4 - source data 2/Figure 4C - source data raw.tiff]
