# Supplementary material for: The Axin scaffold protects the kinase GSK3β from cross-pathway inhibition
Source: eLife. 2023 Aug 7;12:e85444. doi: 10.7554/eLife.85444 (PMC10442075; doi:10.7554/eLife.85444)
Supplement: Supplementary file 1. — Standard errors are from non-linear least squares fits to the initial rate data as described in the Methods. (a) Kinetic parameters for GSK3β reactions with pS45-β-catenin, related to Figures 2–3. a See Figures 2 and 3 for data. The pS9-GSK3β reactions in the absence of Axin did not detectably saturate up to 2 μM substrate (Figures 2 and 3), and only the value of kcat/KM could be accurately determined. No deviation from linearity was observed at 2 μM pS45-β-catenin (the highest pS45-β-catenin concentration tested), suggesting a conservative estimate that KM ≥2 μM. pS9-GSK3β, GSK3β, and GSK3β_S9A were coexpressed with lambda phosphatase. pS9-GSK3β and GSK3β_S9A were incubated with PKA and ATP before use (see Methods). The kcat/KM for λPPase-treated GSK3β is ~5 fold higher than for non-λPPase-treated GSK3β used in previous studies (Gavagan et al., 2020) (see Figure 2—figure supplement 9C and e). (b) Kinetic parameters for PKA reactions, related to Figure 4. (c) KM, ATP values for all reactions, related to Figure S3. (d) Kinetic parameters for pS45-β-catenin reactions with non-PKA treated GSK3β_S9A with and without λPPase treatment, related to Figure 2—figure supplement 9. a λPPase-treated GSK3β_S9A was coexpressed with lambda phosphatase before use (see Methods). Untreated GSK3β_S9A was expressed without lambda phosphatase. (e) Values of kcat/KM for untreated, λPPase-treated, and PKA-treated GSK3β and GSK3β_S9A in reactions with the substrate pS45-β-catenin, related to Figure 2—figure supplement 9. a See (Gavagan et al., 2020) and Figures 2 and 3, and Figure 2—figure supplement 9 for data. λPPase-treated GSK3β and GSK3β_S9A were coexpressed with lambda phosphatase before use. PKA-treated GSK3β and GSK3β_S9A were coexpressed with lambda phosphatase and then incubated with PKA and ATP before use (see Methods). PKA-treated GSK3β is pS9-GSK3β. The kcat/KM value for untreated GSK3β is from previous work (Gavagan et al., 2020). (f) Protein expression plasmids, related to Me [file elife-85444-supp1.docx]

**Supplementary File 1a.** Kinetic parameters for GSK3β reactions with pS45-β-catenin, related to Figures 2-3.^a^

| **Enzyme** | **Reaction** | *k*_cat_ (s^-1^) | *K*_M_ (µM) | *k*_cat_/*K*_M_ (M^-1^s^-1^) |
| --- | --- | --- | --- | --- |
| pS9-GSK3β | –Axin | n.d. | n.d. (≥ 2 μM) | (4.6 ± 0.1) × 10^3^ |
|  | +full length Axin | (2.9 ± 0.3) × 10^-2^ | 0.29 ± 0.08 | (1.1 ± 0.3) × 10^5^ |
| GSK3β | –Axin | (2.8 ± 0.2) × 10^-1^ | 0.33 ± 0.08 | (8.5 ± 2.1) × 10^5^ |
|  | +full length Axin | (3.4 ± 0.1) × 10^-1^ | 0.17 ± 0.02 | (2.0 ± 0.2) × 10^6^ |
| GSK3β_S9A | –Axin | (2.8 ± 0.2) × 10^-1^ | 0.27 ± 0.05 | (9.5 ± 1.8) × 10^5^ |
|  | +full length Axin | (3.3 ± 0.1) × 10^-1^ | 0.16 ± 0.02 | (2.1 ± 0.3) × 10^6^ |

**Supplementary File 1b.** Kinetic parameters for PKA reactions, related to Figure 4.

| **Substrate** | **Reaction** | *k*_cat_ (s^-1^) | *K*_M_ (µM) | *k*_cat_/*K*_M_ (M^-1^s^-1^) |
| --- | --- | --- | --- | --- |
| GSK3β | –Axin | (7.5 ± 0.3) × 10^-3^ | 0.062 ± 0.007 | (1.2 ± 0.1) × 10^5^ |
|  | +full length Axin | (4.4 ± 0.1) × 10^-3^ | 0.25 ± 0.02 | (1.7 ± 0.1) × 10^4^ |
|  | +miniAxin | (4.8 ± 0.4) × 10^-3^ | 0.23 ± 0.05 | (2.1 ± 0.5) × 10^4^ |
|  | +Axin peptide | (6.4 ± 0.5) × 10^-3^ | 0.42 ± 0.06 | (1.5 ± 0.3) × 10^4^ |
| CREB_127-135_ | –Axin | (2.3 ± 0.04) × 10^-2^ | 0.15 ± 0.01 | (1.5 ± 0.1) × 10^5^ |
|  | +full length Axin | (2.3 ± 0.05) × 10^-2^ | 0.14 ± 0.01 | (1.6 ± 0.1) × 10^5^ |

**Supplementary File 1c.** *K*_M, ATP_ values for all reactions, related to Figure S3.

| **Enzyme** | **Substrate** | **Reaction** | *K*_M, ATP_ (µM) |
| --- | --- | --- | --- |
| GSK3β | pS45-β-catenin | –Axin | 5.6 ± 0.9 |
|  |  | +full length Axin | 3.7 ± 1.3 |
| PKA | GSK3β | –Axin | 3.0 ± 0.6 |
|  |  | +full length Axin | 3.3 ± 0.8 |
| PKA | CREB_127-135_ | –Axin | 2.1 ± 0.4 |
|  |  | +full length Axin | 2.1 ± 0.2 |

**Supplementary File 1d.** Kinetic parameters for pS45-β-catenin reactions with non-PKA treated GSK3β_S9A with and without λPPase treatment, related to Figure S12.^a^

| **Enzyme** | **Reaction** | *k*_cat_ (s^-1^) | *K*_M_ (µM) | *k*_cat_/*K*_M_ (M^-1^s^-1^) |
| --- | --- | --- | --- | --- |
| GSK3β_S9A | Not treated | (5.7 ± 0.05) × 10^-2^ | 0.37 ± 0.1 | (1.5 ± 0.5) × 10^5^ |
|  | λPPase-treated | (3.2 ± 0.2) × 10^-1^ | 0.32 ± 0.1 | (9.9 ± 2.2) × 10^5^ |

**Supplementary File 1e.** Values of *k*_cat_/*K*_M_ for untreated, λPPase-treated, and PKA-treated GSK3β and GSK3β_S9A in reactions with the substrate pS45-β-catenin, related to Figure S12.^a^

| **Enzyme** | **Reaction** | *k*_cat_/*K*_M_ (M^-1^s^-1^) |
| --- | --- | --- |
| GSK3β | Untreated | (1.8 ± 0.2) × 10^5^ |
|  | λPPase-treated | (8.5 ± 2.1) × 10^5^ |
|  | PKA-treated | (4.9 ± 0.1) × 10^3^ |
| GSK3β_S9A | Untreated | (1.5 ± 0.5) × 10^5^ |
|  | λPPase-treated | (9.9 ± 2.2) × 10^5^ |
|  | PKA-treated | (9.5 ± 1.8) × 10^5^ |

**Supplementary File 1f.** Protein expression plasmids, related to Methods

| **Plasmid** | **Protein**^a^ | **Expressed Protein** | **Vector**^b^ | **Source** |
| --- | --- | --- | --- | --- |
| pMG024^c^ | GSK3β  Lambda phosphatase (λPPase) | MBP-GSK3β-HA-His  GST_λPPase | pMBP-MG | *This study* |
| pMG071^c^ | GSK3β_S9A  Lambda phosphatase (λPPase) | MBP-GSK3β_S9A-HA-His  GST_λPPase | pMBP-MG | *This study* |
| pES001 | GSK3β | MBP-GSK3β-HA-His | pMBP-MG | *(Gavagan et al., 2020)* |
| pES002 | GSK3β_S9A | MBP-GSK3β_S9A-HA-His | pMBP-MG | *This study* |
|  | | | | |
| pEF073 | Axin | MBP-Axin-His | pMBP-MG | *(Gavagan et al., 2020)* |
| pMG023 | Axin_384-518_ (miniAxin) | MBP-Axin_384-518_-His | pMBP-MG | *(Gavagan et al., 2020)* |
|  | | | | |
| pMG051^d^ | β-catenin CK1α | MBP-β-catenin-His GST-CK1α | pMBP-MG | *(Gavagan et al., 2020)* |
| pEF086 | CREB (127-135) | MBP-CREB_127-135_-His | pMBP-MG | *(Gavagan et al., 2020)* |
|  | | | | |
| pMG026 | Lambda phosphatase (λPPase) | His-λPPase | pBH4 | *This study* |
|  | | | | |
| H_6_-rC | PKA catalytic subunit | His-PKA-rC | pET15b | Addgene #14921 |
|  | | | | |

**Supplementary File 1g.** Plasmids for cell culture experiments, related to Methods

| **Plasmid** | **Protein** | **Expressed Protein** | **Vector** | **Source** |
| --- | --- | --- | --- | --- |
| pES1028 | Axin | Axin-mCherry | pCDNA3.1(+) | *This study* |
| pEK102 | mCherry | mCherry | pCDNA3.1(+) | *This study* |

**Supplementary File 1h.** Antibodies, related to Methods

| **Antibody** | **Source** | **Identifier** |
| --- | --- | --- |
| Anti-GSK-3β (pY216) | BD Biosciences | Cat# 612312; RRID:AB_399627 |
| Anti-GSK-3β (pS9) | Cell Signaling Technology | Cat# 5558; RRID:AB_10013750 |
| Anti-GSK-3β | Cell Signaling Technology | Cat# 9832; RRID:AB_10839406 |
| MBP Tag (8G1) | Cell Signaling Technology | Cat# 2396; RRID:AB_2140060 |
| anti-Phospho-β-Catenin (Ser33/37/Thr41) | Cell Signaling Technology | Cat# 9561; RRID:AB_331729 |
| anti-Phospho-CREB (Ser133) | Cell Signaling Technology | Cat# 9198; RRID:AB_2561044 |
| IRDye 800CW Donkey Anti-Mouse IgG | Li-Cor | Cat# #926-32212; RRID:AB_621847 |
| IRDye 800CW Goat Anti-Rabbit IgG | Li-Cor | Cat# 926-32211; RRID:AB_621843 |
| IRDye 680RD Donkey Anti-Mouse IgG | Li-Cor | Cat# 926-68072; RRID:AB_10953628 |
